# Fluorescence Methods for the Detection of Bioaerosols in Their Civil and Military Applications

**DOI:** 10.3390/s23063339

**Published:** 2023-03-22

**Authors:** Mirosław Kwaśny, Aneta Bombalska, Miron Kaliszewski, Maksymilian Włodarski, Krzysztof Kopczyński

**Affiliations:** Institute of Optoelectronics, Military University of Technology, gen. Sylwestra Kaliskiego 2, Str., 00-908 Warsaw, Poland

**Keywords:** air monitoring, laser-induced fluorescence, bioaerosol, biological particles, lidars

## Abstract

The article presents the history of the development and the current state of the apparatus for the detection of interferents and biological warfare simulants in the air with the laser-induced fluorescence (LIF) method. The LIF method is the most sensitive spectroscopic method and also enables the measurement of single particles of biological aerosols and their concentration in the air. The overview covers both the on-site measuring instruments and remote methods. The spectral characteristics of the biological agents, steady-state spectra, excitation–emission matrices, and their fluorescence lifetimes are presented. In addition to the literature, we also present our own detection systems for military applications.

## 1. Introduction

Biological material emitted to the atmosphere from biogenic sources or intentionally introduced can seriously affect human health and many different environmental processes [1,2,3]. We inhale and exhale air containing thousands of particles such as living, dormant, dead, pathogenic, allergenic, or biologically inert materials [3]. Primary biological aerosol particles (PBAP) or “bioaerosols” may contain bacteria, viruses, fungal spores, pollen, animal and human debris, fragments of leaves, vegetative detritus, fungal hyphae, and biopolymers [4]. Aerosols also contain aromatic hydrocarbons from industrial chemicals and engine exhaust.

Air monitoring is an important element of civil protection. It is used both in early warning systems against a potential bioterrorist act and in determining the air purity in workspaces (production, service, office, hospital rooms, ventilation inlets), residential, public utility, means of transport as well as in the air [5]. A separate problem related to the threat of bioaerosols was the fear of using biological warfare agents. Biological weapons (BWs) are a type of weapon of mass destruction in which the combat charge are pathogenic microorganisms (e.g., anthrax) or viruses (e.g., smallpox virus).

Biodetection is a compromise between time and the amount of information on the specificity of a biological agent. Its types can be divided into the warning, classification, and identification of biological particles. The fastest real-time detection techniques use particle counters and analyzers based on light scattering. Physical methods enabling the classification of biological agents include spectroscopic methods: FTIR [6,7,8,9], Raman [10,11], and fluorescence [12,13,14,15]. Identification is only possible using biochemical techniques (nucleic acid sequence identification, affinity, and the specificity of natural antibodies for detecting antigens).

One of the most important and sensitive methods of the spectroscopic techniques is based on ultraviolet laser-induced fluorescence (UV-LIF). It was originally implemented by the military research community for the rapid detection of biological warfare agents. LIF plays an important role in the detection and classification of biological material in the air [16,17], on surfaces [18,19], in water [20], and in medical diagnosis [21,22]. The construction of biosensors based on UV light-induced fluorescence and elastic scattering began at the end of the 20th century. From 1990 to 2005, advanced detection systems were built and many tests were carried out in both field and laboratory conditions [23,24,25]. Further development of the devices was based on improving the ideas and solutions of previously developed devices. The subject of bioaerosol detection and its development prospects are still very up-to-date and are the subject of review publications [26,27,28]. A number of research groups have constructed detector prototypes to measure the LIF of individual aerosol particles in one or two broadband wavelength channels (266, 355, 375 nm) during the last decades [29,30,31]. In more advanced prototypes, to excite fluorescence, two wavelengths (266, 355 nm) have been used [32,33] or the entire spectrum measured [34]. In the following years, further analyzers were built using the LIF method for the detection of biological analyzers [35,36]. So far, the most technologically advanced instrument is the LIF instrument, which uses fluorescence lifetime measurements in four spectral ranges using two excitation wavelengths (293 and 337 nm) [37]. Fluorescence lidars for BW detection are also technologically advanced [38].

Now, UV-LIF instrumentation has also been commercialized for use in civil research in fields related to atmospheric science and health care. The instruments are used in the study of bioaerosols in the hospital environment [39,40], in school classrooms [41], offices [42], or from humans [43]. They allow for study of the concentration and properties of bioaerosols from long-distance transport [44], in tropical aerosols [45,46], urban aerosols [47,48], or from composting plants [49]. Bioaerosols can also influence cloud formation, precipitation, and climate change [50,51,52,53].

The article presents the history of the development of devices based on LIF, their construction, and principle of operation. In addition to classical instruments using real-time single-particle fluorescence measurements at one or two wavelength excitation, more sophisticated techniques combining LIF with molecular size and shape measurements were demonstrated. An important progress is the construction of instruments that use fluorescence measurements over time.

The general characteristics of biological warfare agents are presented as are the fluorescence characteristics of biological agents in the form of emission–excitation maps, methods, and the results of time-resolved fluorescence studies. Examples of our own solutions for bioanalyzers using the LIF method were also demonstrated.

## 2. Characteristics of Biological Warfare Agents

It is customary to also include weapons based on toxins of biological origin (e.g., botulinum, ricin) as biological weapons (BWs). Biological warfare agents are classified into three main groups by the U.S. Department of Defense: bacterial, viral, and toxin, and there are no known biological agents that are molecular vapors. BWs can be used during an attack on individuals, military units, and civilians. The target of a biological attack can also be homogeneous plant monocultures or livestock farms. The specificity of BW is to cause infectious diseases of people, animals, and plants on a massive scale.

Biological weapons are extremely dangerous, the effects of which are visible only after a long period of time. Anthrax sticks can lie in the ground for up to 40 years, and 2 g of a biological agent can kill 0.5 million people.

Infection with BWs takes place mainly through the respiratory and digestive tracts and open wounds. The majority of BWs are most effective as a finely atomized aerosol that can penetrate deep into the lungs. Penetration into the alveoli is easy when the aerosol particles are 1–5 μm in diameter. BWs come in the form of a dry powder or a liquid suspension. Aerosol droplets can contain from 1 to 50 organisms, and their concentration is in the order of 102 to 106 particles/m^3^.

The diffusion coefficient of microorganisms in air is many orders of magnitude lower than for gaseous molecules. This is important for the spread of diseases if there is negligible turbulent mixing, and the particle cloud takes a long time to disperse. When particles are transported through the air and encounter solid objects, they are deposited due to their greater inertia. This is the primary mechanism by which plant pollen reaches objects. The occurrence of microorganisms (e.g., Bacillus, Micrococcus, Arthrobacter, Staphylococcus, and Brachybacterium) has been found at an altitude of up to 12 km [54]. The influence of atmospheric conditions on the movement of microorganisms is presented in [55,56,57]. Microbes are spread by wind over long distances. A significant impact of the fog carrying microorganisms characteristic of the marine environment inland, over a distance of 50 km, was also observed [58]. Studies on the generic composition of microorganisms at different altitudes have shown that at an altitude of 10 m above the ground, there are different species of microorganisms than at an altitude of 800 m, where the air is already rarefied [59].

All microorganisms are generated by several types of destructive processes. It can be, for example, air movement entraining individual solid particles or water suspensions and separating them from the ground.

An example of the process of creating an infected aerosol from a liquid suspension is sneezing, which is triggered by a sharp contraction of the muscles and leads to the expulsion of air at high speed through the nose. The second example is the dispersion of pollen and spores. Plants have developed complex strategies using grain geometry, grain coverings, and pollen sac linings to overcome the effect of van der Waals forces. Therefore, pollen and spores are effectively broken down, even in light wind.

Of the thousands of pathogenic bacteria and toxins that exist in nature, only about 160 have been identified as harmful to humans, and only 30 are considered likely BWs. Table 1 shows examples of the diseases and their pathogens by potential biological agents.

**Figure 1:**
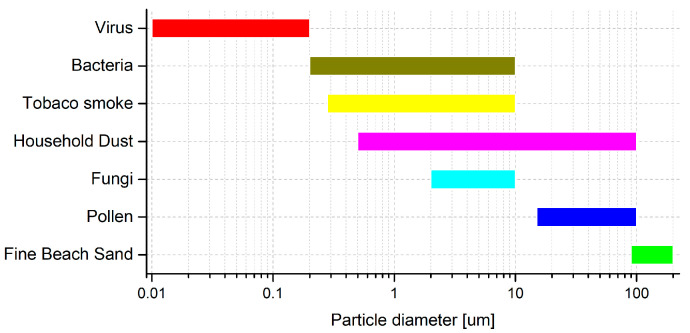
Sizes of the organisms and their interferents.

Particle sizes are the basis for distinguishing different groups of particles (Figure 1).

Man has frequently used biological agents in the fight against the enemy [60,61,62]. One of the first recorded uses of biological warfare occurred in 1347, when Mongol forces are reported to have catapulted plague-infested bodies over the walls into the Black Sea port of Caffa. Infectious diseases brought by the Europeans contributed to the destruction of the Inca and Mayan civilizations.

In 1763, British troops besieged at Fort Pitt during Pontiac’s Rebellion passed blankets infected with the smallpox virus to the Indians, causing a devastating epidemic among their ranks. During World War I (1914–1918), Germany initiated a clandestine program to infect horses and cattle owned by Allied armies on both the Western and Eastern fronts. During World War II, the Japanese used biological warfare agents against the Chinese. Biological weapons have also been used in a few instances in the past by terrorist organizations. In 2001, anthrax-laden letters were sent to many politicians and other prominent individuals in the United States where the letters killed five people and sent 22 to hospital. This event caused many billions of dollars in cleanup, decontamination, and investigation costs.

## 3. Materials and Methods

### 3.1. Materials

The reference materials used throughout this study (Table 2) were prepared as suspensions in water at the Military Institute of Hygiene and Epidemiology (MIHE) in Warsaw [63]. The strains of vegetative bacteria were purchased from the American Type Culture Collection (ATCC.) The samples were prepared from reference stocks and grown in the appropriate liquid media for 18 h at 37 °C in accordance with the procedures consistent with the requirements of the ATCC. Endospores of five Bacillus species were grown, purified, and stored as described by Lewandowski et al. [64]. Candida albicans was grown in liquid yeast-extract-peptone-dextrose growth medium (YPD), and strains of filamentous fungi were cultured in malt extract agar medium (MEA) [65]. The water suspension of the bacteria, endospores, fungi, and pollens were placed in 1 cm thick quartz cuvettes (OD600 = 0.1–0.2) and their spectra and fluorescence decay times were measured. During the measurements, the suspensions were stirred using a magnetic stirrer. The front-surface fluorescence measurement method was used to avoid the internal filter effect caused by the absorption of the emitted radiation.

### 3.2. Measuring Apparatus

**Figure 2:**
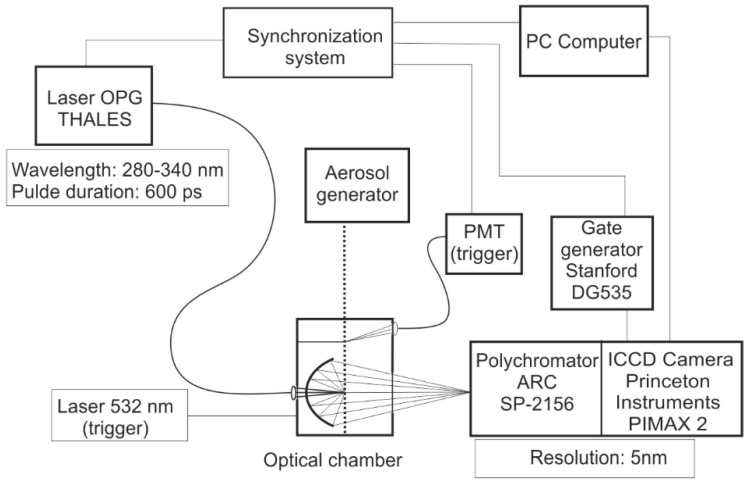
Scheme of the system for measuring the spectra of single particles.

(a) Fluorimeter FS 900 (Edinburgh Instr., Livingston, Scotland) for measuring the excitation–emission matrices (EX-EM);
(b) Ultraviolet aerodynamic particle sizer (UVAPS, model 3314, TSI Inc., St. Paul, MN, USA) for the real-time measurement of single particle’s aerodynamic diameter and fluorescence intensity;
(c) ASPECT (Bristol Industrial & Research—BIRAL, Bristol, UK);
(d) VeroTect^TM^ (Bristol Industrial & Research—BIRAL, Bristol, UK);
(e) EasyLife System (Photon Technology International, Birmingham, NJ, USA) with excitation of 280 and 340 nm for the fluorescence measurements of the suspensions and solid particles. Fluorescence excitation was realized using nanosecond impulse LEDs (Edinburgh Instruments, Livingston, Scotland);
(f) Compressed-air nebulizer Monsun2 MP2 (Medbryt, Warsaw, Poland);
(g) Small scale powder dispenser (TSI Inc., Shoreview, MN, USA);
(h) To measure the fluorescence spectra of single particles of aerosols, our own system was used, as shown in Figure 2.

The Thales laser is described in Section 5 in this article. Principal component analysis (PCA) was performed using the SIMCA-P program from Umetrics (Ulmea, Sweden).

## 4. Methods and Results of Measurements of Biological Materials

### 4.1. Fluorescence Emission–Excitation Matrices (EM-EX)

**Figure 3:**
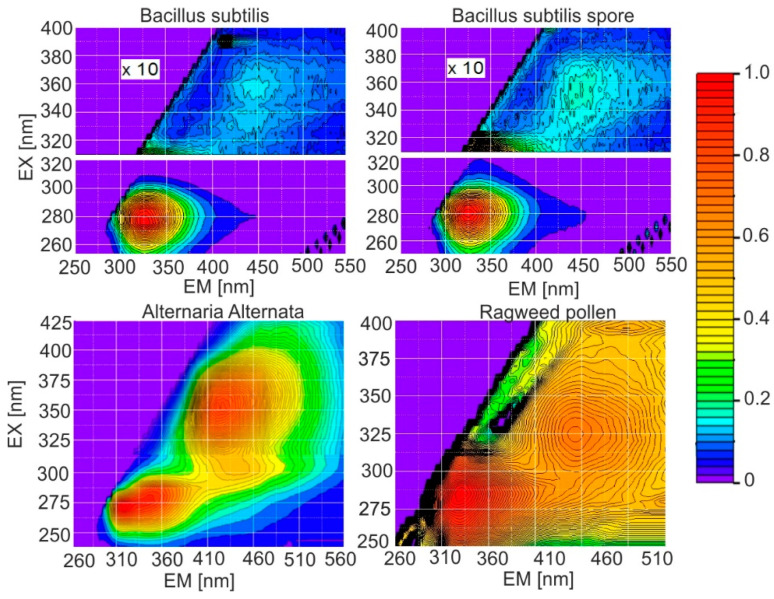
EX-EM matrices of the bacteria and their interferents.

In the case of the presence of many fluorophores, as occurs in microorganisms, the best method of full spectral characterization is emission–excitation matrices. This is a true spectral fingerprint. The first EM-EX measurements of the selected biological materials were described in [65], and now the current database contains about 80 different biological substances and their interfering substances. In the natural environment, bacteria may appear in the same airborne particle with different interferents such as fungi, pollens, cellulose, lignin, humic and fulvic acids, chlorophyll, and dust. Example matrices of the selected bacteria and their interferents are shown in Figure 3.

**Figure 4:**
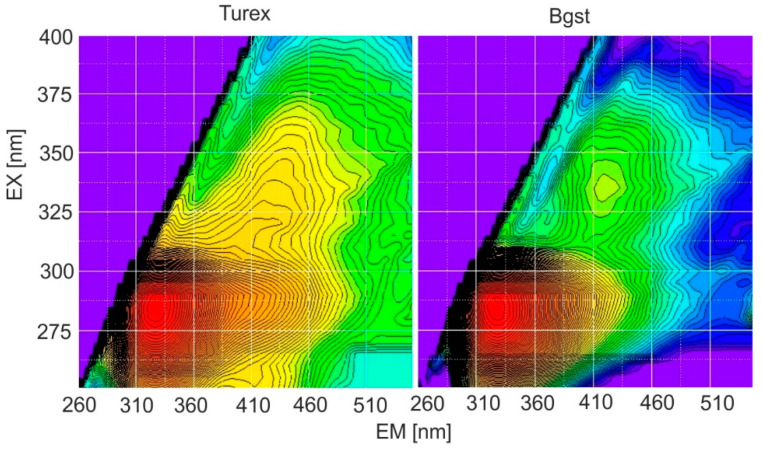
EM-EX matrices of the technical spores.

Spore forms, compared to vegetative bacteria, have much more intense fluorescence in the 350–370 nm region (II region). EX-EM matrices enable the process of sporulation or the death of live bacteria. In practice, the biological warfare material will be a mixture of spores, live and dead cells, admixtures of stabilizers, and culture media. Technical preparations used to test various BW analyzers have even stronger fluorescence bands in the second region (Figure 4).

Many research groups have been involved in the analysis of endogenous biological fluorophores [66,67,68,69,70]. The source of endogenous fluorescence in cells and biological tissues are aromatic amino acids used to build proteins and coenzymes. Of the 20 amino acids from which proteins are built, only tryptophan (TRP), tyrosine (TYR), and phenylalanine (PHE) fluoresce in the 250–290 nm range (range I). The excitation and emission maxima in solution are about 255 and 282 nm for phenylalanine; 275 and 303 nm for tyrosine, and 280 and 350 nm for tryptophan. The second group of fluorescent endogenous compounds includes the following coenzymes: nicotinamide adenine dinucleotide (NADH), flavin adenine dinucleotide (FAD), and flavin mononucleotide (FMN).

These coenzymes cooperate with oxyreductase and their task is to transfer protons and electrons. NADH exhibits strong fluorescent properties, having absorption and emission maxima at 340 and 460 nm wavelengths, while the oxidized form of the coenzyme NAD+ does not fluoresce. FAD and FMN coenzymes absorb light with a wavelength of about 450 nm, and emission occurs at a wavelength of about 530 nm. Spore forms, compared to vegetative bacteria, have much more intense fluorescence in the 350–370 nm region (II region). EX-EM matrices enable the process of sporulation or the death of live bacteria. In practice, the BW material will be a mixture of spores, live and dead cells, admixtures of stabilizers, and culture media.

**Figure 5:**
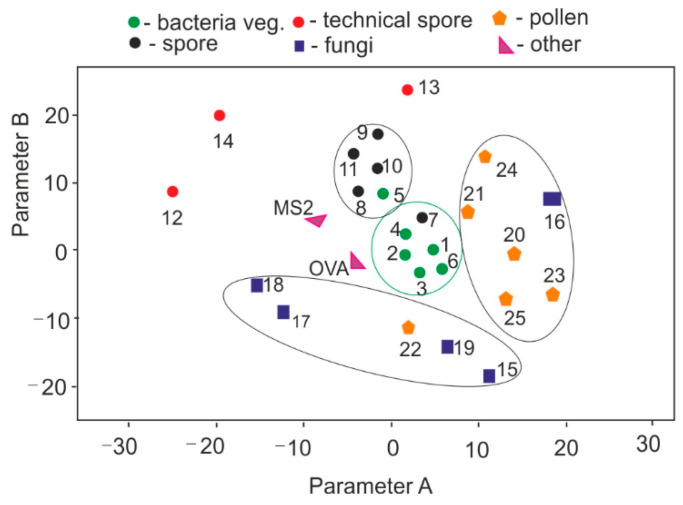
PCA of the EM-EX of the biological materials (description as in Table 1).

Figure 5 shows the analysis of the EM-EX maps of the examined biological materials using the principal component analysis (PCA) method [71]. Practically, the PCA results are interpreted in such a way that the farther apart the points representing the individual substances are, the lower their degree of similarity. The PCA method has been used in the analysis of bioaerosols since 2001 [72]. The collections of pure vegetative bacteria and their spore forms were the most similar. It can be seen that the technical disputes changed the fluorescent properties. Separate groups were pollen and fungi, which have very different characteristics. EX-EM mapping methods and PCA distinguished the individual substances well. This method, however, requires a very long time and a high concentration of test substances and cannot be directly applied to real-time measurement systems.

### 4.2. Fluorescence Lifetime Measurements

The fluorescence lifetime is an important parameter that characterizes the fluorophore and extends the ability to distinguish weak emission signals from the surrounding background. Time-resolved fluorescence is commonly used in the study of macromolecules, mainly proteins, as well as for the characterization of other biological systems [73]. The data obtained by this method often provide more information about the molecular structure than the fluorescence spectra.

This technique is used to measure the resonant energy transfer, fluorescence quenching, interaction of fluorescent probes with proteins, DNA, or membranes, decay anisotropy and rotational state correlation times, lifetime distributions, and multi-exponential decays. The fluorescence lifetime of biofluorophores is very sensitive to their micro-environment in the protein and makes it possible to discriminate between different biological molecules. Times to be expected for fluorescence range from ps to ns and for phosphorescence to μs. Each of these ranges has different technical requirements and presents different measurement challenges. Various methods of time-resolved laser spectroscopy are used to determine the fluorescence lifetime. Currently, the most popular methods of measuring this parameter include:

- Measurement method in the frequency domain, the so-called modulation-phase method (frequency domain);
- Time domain measurement methods.

**Figure 6:**
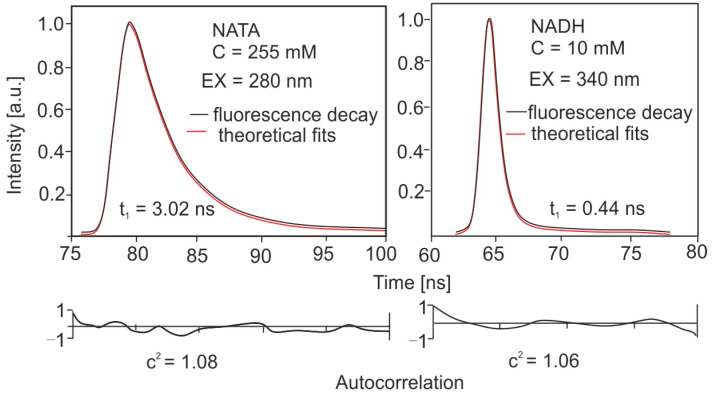
Fluorescence decay curves of NATA and coenzyme NADH.

The classic method, time correlated single photon counting (TCSPC), is characterized by very long measurement times, up to several hours, and during the irradiation, bacteria are degraded. The stroboscopic method [74], developed in the 1990s, allows for the measurement of the fluorescence lifetime in about 10–20 s. This method has also been implemented to measure the lifetime of bacteria and biological interferents [75]. A very important application is the study of the lifetime of chemical or biological compounds. Figure 6 shows examples of the decay of the fluorescence curve for tryptophan methyl ester (NATA) and the NADH coenzyme.

**Figure 7:**
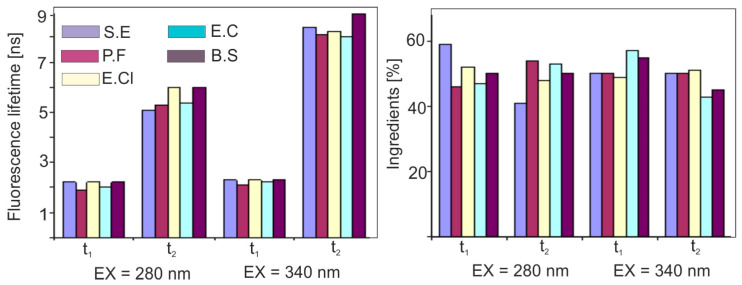
Fluorescence decay characteristics for vegetative forms of bacteria (S.E—*Staphyllococcus epidermis*, E.Cl—*Enterobacter cloacae*, E.C—*Escerica coli*, B.S.—*Bacillus subtilis*, P.F.—*Pseudomonas Fluorescens*).

The NATA and NADH standards are characterized by mono exponential decay and their values are consistent with the literature values determined by other methods. In addition, the signal level is very high with a low noise value at the same time. A very high degree of fit of the theoretical and experimental curve can also be observed, which shows that the method used is reliable. The stroboscopic method is perfect for measuring the lifetime of biological compounds. Using the stroboscopic method, fluorescence time measurement takes only a dozen or so seconds. Figure 7 presents the results of our own research on the lifetime of the selected bacteria.

### 4.3. Measurements of the Size and Shape of Bioaerosol Particles

The fluorescence does not provide sufficient information for the clear and reliable identification of airborne species and only allows for a rough, real-time discrimination of potential intentionally-released biological warfare agents from interferents. For many years, instruments that measure the aerosol size distribution have been available. However, the addition of valuable shape information has enabled a much deeper understanding of the particle populations. Since most particles, either naturally occurring or manmade, are not perfect spheres, particle shape is an important parameter that can classify the particle species. The first device developed by Kaye has been commercialized by BIRAL as an instrument called ASPECT [76,77,78]. Using BIRAL’s ASAS Technology, ASPECT is a particle analyzer combining the size and shape characterization of aerosols. With a unique method of laser light scattering, the instrument performs a rapid analysis of the transient spatial intensity distributions of scattered light from single particles.

**Figure 8:**
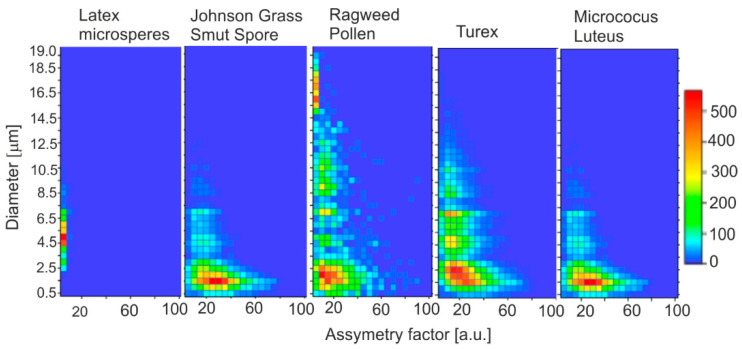
Particle size and size distribution and the SEM image of the chosen particles.

The principle of operation of the instrument is described in more detail in Section 5. Using the optical size and shape analyzer, we found a good correlation between the light scattering properties and the particle features determined by scanning electron and fluorescence microscopy [79]. Additionally, HCA (hierarchical cluster analysis) offers fast and continuous bioaerosol classification. These data could be significant in environmental monitoring, climate research, and industrial settings. Example characteristics obtained using the ASPECT device are shown in Figure 8.

**Figure 9:**
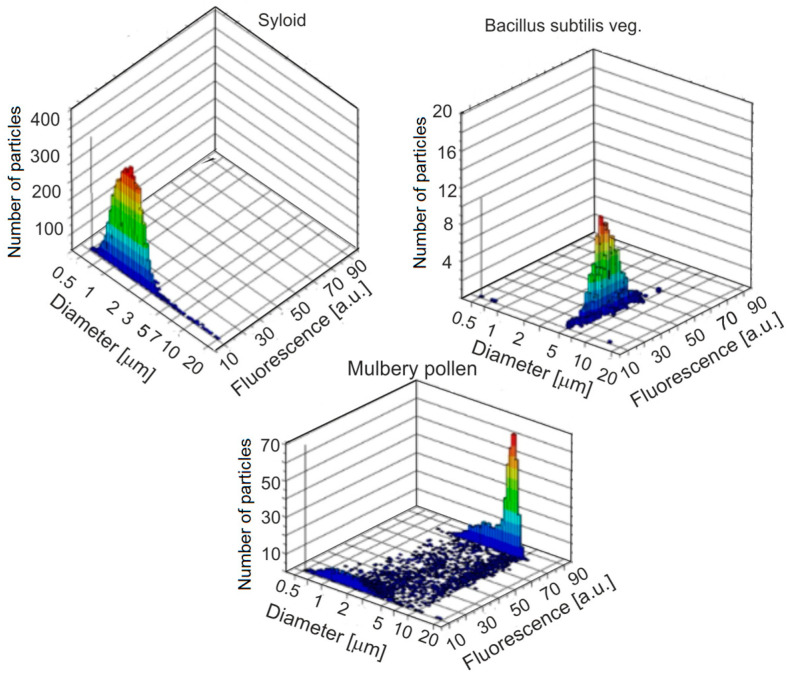
Aerosol particle characteristics measured with a UV-APS analyzer.

One of the most important instruments for studying the fluorescence and size of individual particles of bioaerosols in real-time is UV-APS, which was developed by the Canadian company TSI Inc. The instrument is often used as the standard and reference in the development and construction of various particle analyzers. Figure 9 shows examples of the measured fluorescence characteristics and aerosol particle diameters.

### 4.4. Single Particle Spectra

**Figure 10:**
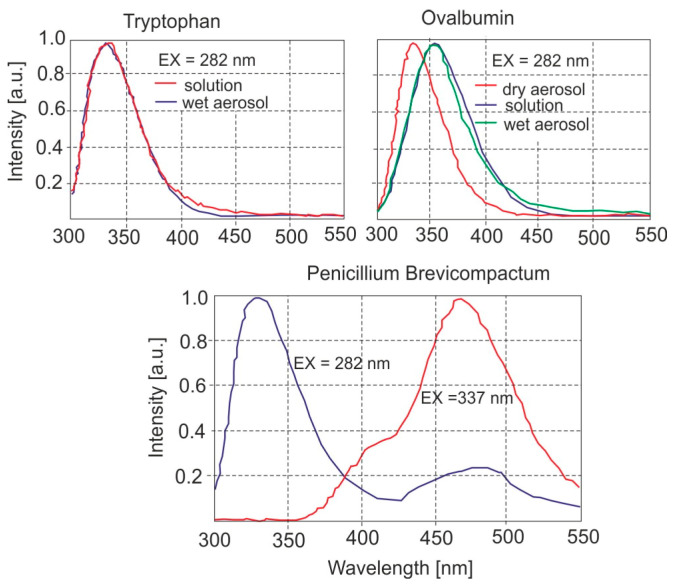
The single-shot spectra from individual particles.

The single-shot, fluorescence spectra from the individual particles of primary fluorophores are presented in Figure 10. Chemical compounds were dissolved in water and the solutions were aerosolized with a compressed-air nebulizer. Emission maxima from different biological materials are not necessarily the same in an aerosol as in solution because the aerosols are often dry or somewhat dry.

## 5. Review of Instrumentation

The construction of biosensors based on UV light-induced fluorescence and elastic scattering began at the end of the 20th century. The idea of building the first analyzers of biological agents consisted in combining the fluorescence technique, commonly used in liquid flow cytometry, and the aerodynamic method of particle size measurement. The system measures the number of particles in the flowing aerosol as a function of scattering and fluorescence. The aerodynamic method of measuring the particle diameter values is commonly used for the analysis of aerosol particle distribution with high resolution in the diameter range of 0.5–15 µm.

**Figure 11:**
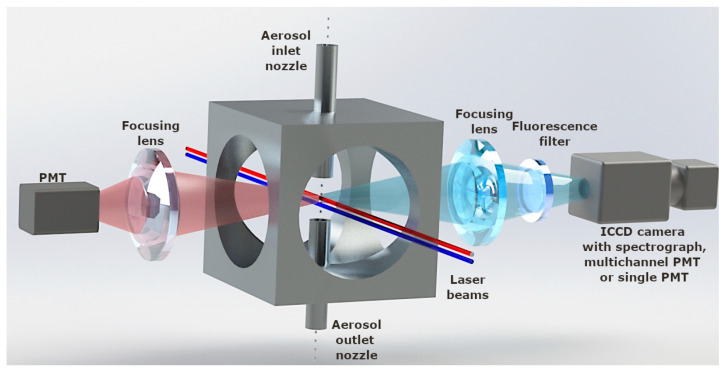
The idea of single particle measurements.

The aerosol particles are passed through a small nozzle, causing them to rapidly accelerate in the air stream. Small particles in the jet accelerate almost like the air stream, and larger particles leave the nozzle later due to their greater inertia. Particle velocities are determined at the exit of the nozzle by measuring their time-of-flights on a specific path controlled by laser radiation and then converting them into particle diameters. The principle of operation of liquid flow cytometry does not consist in passing a liquid stream containing the tested particles through a focused laser beam. The jet is surrounded by a layer of clear liquid that keeps the sample flowing through the radiation focus. Fluorescence from the biological particles is measured using photomultipliers equipped with optical filters or a spectrometer with an ICCD camera (Figure 11).

Flow cytometers enable high-volume single-particle data collection and the real-time characterization of populations of biological particles. The classical method uses fluorescent antigens that form specific complexes with the analyzed antibodies. For the characterization of bacteria suspended in an aerosol, this solution is impractical, and only the fluorescence of endogenous fluorophores contained in the bacteria is determined. Table 3 provides an overview of the bioaerosol detection instruments.

The first measurements of the fluorescence of individual single-particles from *Bacillus anthracis* were conducted by the U.S. Army Research Laboratory (ARL) [80]. The system with a 488-nm laser was used to measure the LIF spectra of atmospheric aerosol [35,81,82] and laboratory-generated particles [83,84].

The Canadian Defense Research Establishment, in cooperation with TSI, Inc. (Shoreview, MN, USA), developed the first commercially available fluorescence-based bioaerosol detectors—the UV-APS and FLAPS for military applications [85,86,87,88,89,90].

MIT Lincoln Laboratory (LL, Lexington, MA, USA) developed a 266-nm-based fluorescence and elastic scattering detector, BAWS (biological-agent warning sensor) [91]. The sensor was used as the trigger on the joint biological point detection system, (JBPDS0) [92,93].

The Naval Research Laboratory (NRL) constructed an elastic scattering-cued fluorescence sensor at 266 nm [25,94]. Cooperation between the LLNRL and the Edgewood Chemical and Biological Center led to the construction of the Rapid Agent Aerosol Detector (RAAD), which consists of 355-nm polarized elastic scattering, and dual fluorescence excitation at 266 and 355 nm [15]. Detection of the fluorescence of individual aerosols with two excitations and broad band emission channels [34] has been shown to better discriminate against common interferents [15]. An even better classification of bioaerosols is possible with a dual excitation system with 32-channel spectral resolution [84]. The University of Hertfordshire and U.K. Defense Ministry developed and commercialized, by BIRAL, the fluorescence and light-scattering instrument WIBS [33,78,98,99], and later VeroTect^TM^ [100].

Similar in design to the WIBS is the multiparameter bioaerosol sensor (MBS) with enhanced spectral resolution and morphological information [101,102]. A xenon lamp (wavelength 280 nm) was used to excite the fluorescence and the fluorescence signal (310–638) was detected over eight channels (310–638 nm) by a multichannel photo-multiplier. A dual CMOS linear array recorded the spatial effects of elastic scattering.

Another modification of the WIBS analyzer is the commercial SBS (spectral intensity bioaerosol spectrometer) [103]. It is a commercial LIF instrument built on the optical block of the WIBS. Excitation pulses from the filtered xenon flash-lamps were centered at 285 and 370 nm and the emission spectra fell into 16 channels (300–720 nm). The SIBS is characterized by a significantly increased spectral resolution relative to WIBS.

Real real-time observations of the total pollen and grass pollen concentrations were performed using the Rapid-E analyzer. The PA-300 (Plair SA, Geneva, Switzerland) [104,105] analyzer was described by Kiselev et al. [106,107]. Rapid-E consists of a blue laser (400 nm) used to produce time-resolved scattering patterns across 24 detectors at different angles.

## 6. Characteristics of Selected Analyzers for the Detection of Bioaerosols

### 6.1. UV-APS

The device most often used as a model or reference in relation to other devices built is the UV-APS (in the military version FLAPS). So far, a very large number of aerosol studies have been carried out using this instrument in both indoor [44,45,108,109] and outdoor analysis [110,111,112,113] to investigate the viability and dynamics of airborne particles [85,87,114,115].

In the first prototype, a He-Cd laser with a wavelength of 354 nm and a power of 30 mW was used as the excitation source; the next versions contained the following lasers: He-Cd (325 nm, 30 mW) and Nd:YAG (355 nm). Pulsed 355-nm light from a Nd:YAG laser excited the fluorescence and the integrated intensity from 420 to 575 nm was measured [116].

The first laboratory tests showed that the upper limit of detection was about 60 particles/cm^3^. In addition to the fluorescence level of individual particles, their aerodynamic diameter was determined, and the combination of these values allowed for the discrimination of different types of biological agents. This device enables the real-time measurement of the aerodynamic diameter, diffuse light intensity, and aerosol fluorescence. The tested aerosol is sucked from the environment through a nozzle with a constant flow rate of 1 L/min. Around the mainstream, a protective jacket is created from a stream of filtered air flowing with a capacity of 4 L/min. Such a system ensures the precise focusing of the bioaerosol in the laser beam. Aerosol analysis takes place in the measuring chamber. The signal coming from the scattering of light on the particle is detected using a photomultiplier. The measurement of the aerodynamic diameter is based on the analysis of the particle’s time-of-flight between two laser beams emitting a red light with a wavelength of 655 nm and a power of 30 mW. The larger the particle, the longer the time-of-flight. The measurement of the fluorescence of individual particles distinguishes the biological material from non-biological. Fluorescence is excited at a wavelength of 355 nm, and the emission is recorded using a photomultiplier in the range of 430–580 nm. The UV laser pulse is triggered based on the time-of-flight measurements, which allows for the determination of the appropriate fluorescence. Most particles are between 0.7 and 3 μm in size, and the average aerodynamic diameter is about 1.5 μm. The examples of the analysis of the size distribution and fluorescence level of various aerosol materials, determined with the UVAPS instrument, presented in Figure 10, indicate a fundamental difference in the emission of these materials. The UV-APS reports fluorescence in a single emission band, with the overall particle discrimination generally poorer than for multichannel instruments.

### 6.2. WIBS

In the WIBS system, pulsed xenon lamps are also used as the excitation source. An example of such solutions are the WIBS1 [117] and WIBS2 [118] prototypes. WIBS1 uses two xenon modules (Perkin Elmer RSL3100) built-in quasi-collimation optics. Their repetition rate is 50 Hz, operates at 2–3 Hz, maximum flash energy of 40 mJ, and a flash duration of 1 ms. The xenon lamps are mounted on either side of the scattering chamber and orthogonal to the axis of the lamps are two fluorescence detectors.

Elastic scatter from a 635-nm laser is used, two xenon flashlamps are filtered to produce narrow excitation wavebands centered at 280 and 370 nm, and two wideband photomultiplier detection channels (310–400 nm and 420–650 nm) provide three channels of fluorescence detection.

The aerosol flow of 10 l/min is drawn from the ambient environment through the central chamber. The WIBS1 sensor records the intrinsic fluorescence from an ensemble of particles simultaneously. The WBS2 sensor overcomes the significant limitations and provides single-particle fluorescence detection. Around a central optical chamber, a CW 660 nm diode laser (or 635 nm laser) is arranged and used to determine the particle size and asymmetry of particles as well as two pulsed xenon sources and two fluorescence detection channels. The aerosol is drawn from the atmosphere via a laminar-flow delivery system with the flow of 4.0 L/min. Each particle entering the chamber produces a scattered light and the size is determined. Particles greater than about 1 μm in size initiate the sequential firing of xenon source.

WIBS2 uses two Hamamatsu L9455 xenon modules (Hamamatsu Photonics K.K., Hamamatsu, Japan) with a maximum repetition rate of 126 Hz. The sensor has the ability to differentiate between various biological and non-biological airborne particles down to 1 μm. The most recent version is WIBS4 [16,118,119,120]. Fluorescence emitted by a given particle after each excitation pulse is detected simultaneously using two PMT detectors. The first PMT is optically filtered to detect the total intensity of fluorescence in the range 310–400 nm and the second PMT in the range 420–650 nm. Therefore, for every particle that triggers xenon lamp flashes, Xe1 produces a signal in the FL1 (310–400 nm) and FL2 (420–650 nm) channels, whereas Xe2 produces only a signal in the FL3 (420–650 nm) channel because the elastic scatter from the Xe2 flash saturates the first PMT. Elastic scatter from a 635-nm laser or 660 nm laser diode is used to determine the particle size and asymmetry, two xenon flashlamps are filtered to produce narrow excitation wavebands centered at 280 and 370 nm, and two wideband photomultiplier detection channels (310–400 nm and 420–650 nm) provide three channels of fluorescence detection.

The wideband integrated bioaerosol spectrometer (WIBS) is a widely used, commercial, three-channel LIF spectrometer developed by the University of Hertfordshire (UH), now licensed to and manufactured by Droplet Measurement Technologies (DMT; Longmont, Colorado). Several non-commercial WIBS versions have been produced with slightly different optical and electronic configurations, and DMT has manufactured two commercial models (4A and NEO).

Savage et al. [16] presented a systematic characterization of the WIBS4A instrument using 69 types of aerosol materials including a representative list of pollen, fungal spores, and bacteria as well as the most important groups of non-biological materials reported to exhibit interfering fluorescent properties. Broad separation can be seen between the biological and non-biological particles directly using the five WIBS output parameters and by taking advantage of the particle classification analysis introduced by Perring et al. [121]. Hernandez et al. [122] presented a summary of more than 50 pure cultures of bacteria, fungal spores, and pollen species analyzed by the WIBS with respect to the fluorescent particle type.

### 6.3. ASAS and VeroTect^TM^

The combination of fluorescence analysis and light scattering on individual air particles has become an important tool that can characterize, classify, and in some cases, identify biological particles. This technique allows for non-invasive, non-destructive analysis in real-time and is widely used in the study of environmental pollution and the detection of deliberately introduced combat pathogens.

Since most particles either naturally occurring or manmade are not perfect spheres, the particle shape is an important parameter in which the particle species can be classified. For spherical particles illuminated by circularly polarized radiation, the scattering intensity does not change in different azimuthal planes for the entire range of polar angles. However, the presence of non-spherical particles breaks this symmetry, and the scattering for the selected azimuthal plane is dependent on the orientation of the particle. This approach was developed by Kaye [24,76]. In the original system, three photodiodes were arranged symmetrically at 40° in a circle around the *z*-axis of the incident beam of circularly polarized radiation.

The detectors collected light scattered by single particles flowing in laminar motion. The signals recorded by azimuthal detectors allowed for the distinction between the spherical and non-spherical particles. The aim was to distinguish, in real-time, between spherical droplets of potential contaminants. The system was modified several times in order to increase the measurement range of particle diameters to 1 μm and the value of the polar angle.

This setup was the starting point for BIRAL’s particle size and aspect ratio analyzers. The technology is known as ASAS (aerosol size and shape). The measurement of the number of particles, size, and aspect ratio is based on ASAS technology. It consists of the analysis of light scattered on individual particles. The addition of valuable shape information has enabled a deeper understanding of particle morphology. One of the simplest methods of reducing the shape-related light scattering data is the asymmetry factor *A_f_,* a measure of the azimuthal variability of the scattered radiation. The three azimuthal detectors *E*_1_*, E*_2_, and *E*_3_ provide information on the shape of the particles, and the scattering data measured on the single detector *E*_4_ relates to the particle sizes. A spherical particle such as a drop of liquid scatters the radiation to three azimuthal detectors. Elongated particles (e.g., fibers) tend to align themselves parallel to the flow direction and thus scatter, mainly horizontally, to one *E*_2_ detector. Particles with other morphologies have intermediate scattering distributions in relation to the extremes above-mentioned. *A_f_* is expressed by the following formula:

$$A_{f}=\sqrt[{k}]{\overset{i=n}{\underset{i=1}{\sum }}\;{\left(E_{s}-E_{i}\right)}^{2}}/E_{s}$$

where *E_s_* means *E*_1_ *+ E*_2_ *+ E*_3_, and *k*—is a constant chosen to reach the maximum value of *A_f_*.

For spherical particles, *A_f_* is zero, and the orientation in one direction is 100. Despite the simplicity of this method, estimating the value of *A_f_* is effective in the differentiation of real-time morphology.

A combination of measurements of the fluorescence intensity, size, and aspect ratio of bioaerosol particles was used in BIRAL’s VeroTect^TM^ system. The instrument enables real-time, generic biodetection, characterizes the ambient background aerosol, and detects changes associated with the presence of biological threats. Fluorescence is measured on a bulk sample of the aerosol in two ranges: 330–650 and 420–650 nm, respectively. The system uses an innovative light source centered at 280 nm, the optimum excitation wavelength for biodetection. The size range of measured particles is 0.5–15 µm, flow rate—2 L per minute, and the max. particle throughout is 20.000 per second. Characterization by size, concentration, and particle shape is a powerful means of distinguishing between the potential threat and benign interferents (pollen, diesel fuels).

### 6.4. BARDet

Semiconductor laser diodes are also beginning to be used in bioaerosol detection systems. An example is the airborne particle flow detector BARDet (bioaerosol detector) built at the Institute of Optoelectronics (IOE) of the Military University of Technology (MUT) in Warsaw.

The scattering and fluorescence intensity of individual particles are measured in seven bands [32]. The fluorescence excitation source is a CW 375 nm laser (Coherent CUBE CW). The aerosol is drawn through the inlet nozzle and focused by a flow of filtered shielding air, and the flow is perpendicular to the plane of the laser beam. The aerosol and focus stream flow rates are approximately 0.15 and 2 L/min, respectively. Back and forward stray light is detected with two PMT detectors (Hamamatsu, H6780, Japan), which for backward and forward scattered light measurements are mounted at 35° and 145° laser beam angles, respectively, and the fluorescent signals are collected at 90° angles to the excitation laser beam. Fluorescence signals are collected by a spherical mirror and reflected to a polychromator integrated with the 32-channel PMT. The compact design allows it to be mounted on a mobile platform.

The artificial neural network (ANN) method is used to analyze data obtained from measurements using the BARDet device. A total of 48 different aerosols including bacteria, pollens, fungi, spores and non-biological substances have been analyzed and it was found that almost every particle could be properly classified [123]. At the Institute, the device is used as a reference in the field tests of bioaerosols. The prototype of the device is at the disposal of the Field Biological Laboratory.

### 6.5. The Fabiola Project

The next stage in the development of the LIF method was the use of different fluorescence lifetimes of individual biological media, which was implemented in the FABIOLA program [37,124]. Constructed by a consortium of many international centers, the analyzer has successfully passed laboratory and field tests. The device consists of the following components:

- Particle segregation chambers [125];
- OPG laser for fluorescence excitation with 293 nm and 337 nm wavelength radiation [126];
- Optical system for the acquisition of emission signals in the spectrum of the four spectral ranges;
- Software enabling the quick analysis and grouping of signals using the PCA method.

**Figure 12:**
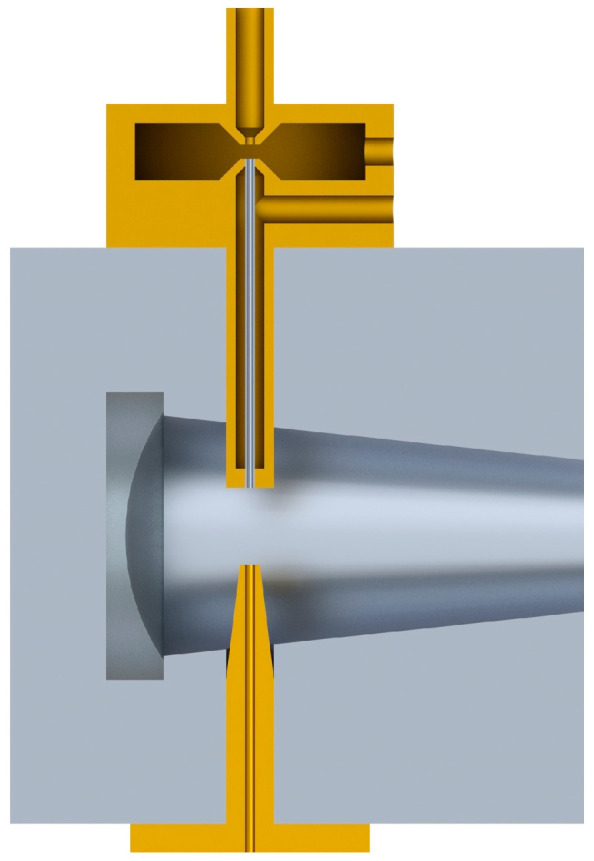
Construction of the particle concentrator and view of the chamber.

The measuring chamber is the heart of the system; it also acts as a particle concentrator (Figure 12).

In the central part of the chamber, air with biological particles flows in a laminar flow. The flow rate is 2 dm^3^/min. In the particle concentrator, the air stream is divided into two streams with flows of 1.8 dm^3^/min and 0.2 dm^3^/min, respectively. The main stream, containing particles with diameters below 1 μm, is removed from the system. The secondary stream, containing particles with diameters of 1–10 µm, continues to the nozzle. Theoretically, the particle concentration factor (Q_tot_/Q_sec_) is 10. This stream is additionally surrounded by an external, additional air stream with a flow of 1.6 dm^3^/min in order to stabilize it. Upon reaching the nozzle, the particles are rapidly accelerated. They encounter laser radiation with a wavelength of 650 nm, followed by OPG laser radiation. A laser diode with red radiation acts as a trigger for the UV laser. The fluorescence radiation is focused on an elliptical mirror. At the first focus of the ellipse, the particle is in contact with the fluorescence excitation radiation; at the second focus of the ellipse, the exit slit is placed, and the radiation is focused.

**Figure 13:**
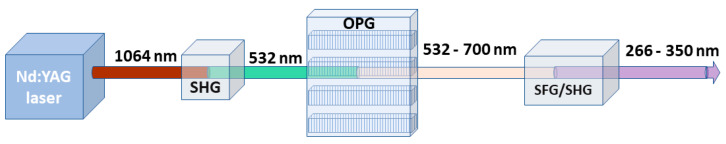
Schematic of the OPG laser.

The OPG laser constructed by Thales (Figure 13) consists of a main oscillator with an amplifier (λ = 1064 nm, E_imp_ = 61 µJ, ν = 1 kHz) and radiation conversion blocks [127]. Second harmonic generation takes place in a KTP crystal with a length of 15 mm. The temperature of the crystal is stabilized by a thermoelectric cooler. At the exit from the crystal, radiation with a wavelength of 532 nm is obtained, which then, in the crystal of lithium niobate doped with magnesium (PPMgLN), generates radiation in the range of 590–700 nm.

The conversion of this radiation to the range of 290–350 nm (SGH) takes place in the BBO crystal. The conversion of radiation from the 580–700 nm range to the 280–300 nm range is generally done by generating a summation frequency. The laser radiation is introduced into the optical fiber through a filter made of UG11 glass, transmitting only UV radiation. The laser has been modified so that it is possible to generate both beams simultaneously. The 337 nm beam was delayed by a fiber optic line to arrive at the chamber 80 ns later than the 293 nm beam.

**Figure 14:**
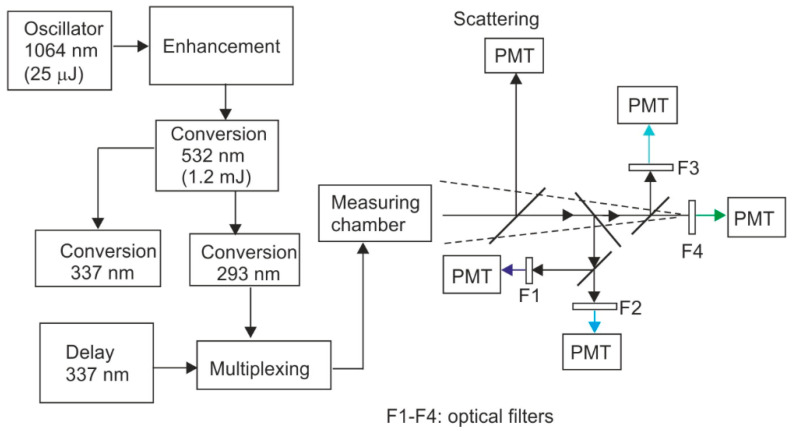
Schematic diagram of the four-channel bioanalyzer: F1–F4—optical filters.

Limitations of the size of the equipment and the sensitivity of the apparatus forced the choice of two excitation lines lying in the TRP and NADH emission bands of bacteria (293 and 337 nm). The number of emission bands at which the emission decay was tested was limited to four due to sensitivity. The scheme of the bioanalyzer is shown in Figure 14. The decay of fluorescence is measured in successive channels. The measurement of the scattered laser radiation is made in a photomultiplier tube, and in the next four emission channels, the fluorescence decay over time is measured. In front of each photomultiplier, there is an appropriate interference filter for a given range of wavelengths. Diffuse radiation makes it possible to measure the particle diameter.

**Figure 15:**
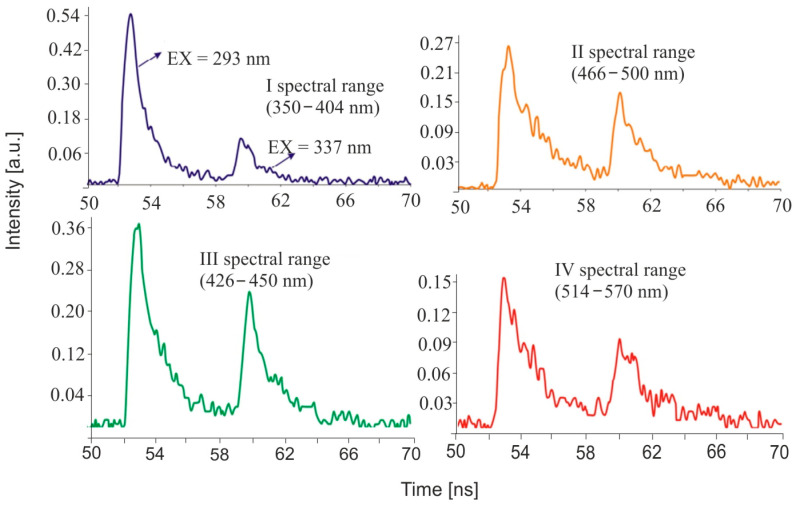
Emission characteristics of a single particle of BG.

Figure 15 shows an example of the emission characteristics derived from the excitation of a single particle of BG material.

On the ordinate axis, the normalized intensity values correspond to the actual intensity ratios in the individual channels. Fluorescence emission bands are strongest in the wavelength range of 350–404 nm and decrease with increasing wavelength. The areas under the individual decay curves correspond to the integrated fluorescence intensity. In this way, two important emission parameters are obtained: the intensity levels for different spectral ranges and the emission lifetimes. The measurements are very fast, and every single particle that enters the chamber in the flowing air stream is analyzed. The system was tested in laboratory conditions in the chamber (Paris) and on the training ground in Sweden. Appropriate differentiation between tested simulators and BW interferers was achieved.

## 7. Stand-Off Detection of Bioaerosols

Compared to in situ analyzers, stand-off detection is less frequently used. Lidar is one of the very few promising methods in terms of long-range stand-off airborne biological particles. Initially, lidars were used in atmospheric research [128,129], and over time, this technique was adopted for military applications [130,131]. Scattering lidar is the most basic representative of the family. LIF-lidar methods are based on the fluorescence emission of biological particles. This technique is one of the very few promising methods in terms of the long-range detection of airborne particles. Standard remote sensing systems are used for atmospheric composition, environmental research, national security and agricultural monitoring. These systems are designed for both ground and air applications [132,133,134,135,136].

The advantage of remote methods over point detectors results from the possibility of observing large areas, in the order of tens of kilometers, with a high spatial resolution of several meters and without the need to physically reach the target. Quantitative, spatially resolved measurements of bioaerosol concentration and properties are primarily provided by active methods of light detection, and distance determination enables the measurement of the bioaerosol concentration and properties [137]. Lidars have been used to monitor various types of measurements [138,139,140]. The LIF-lidar technique mainly uses high-power pulsed UV lasers, usually with an excitation wavelength of 266 or 355 nm (e.g., with a pulsed Nd:YAG laser).

A typical new generation lidar was presented in [38]. The device is based on two pulsed UV laser source operating at 266 nm and 355 nm wavelengths (4th and 3rd harmonic of Nd:YAG and Q switched, respectively). Range resolved fluorescence signals are collected in 28 channels of a compound PMT sensor coupled with a Czerny–Turner spectrograph. System tests were carried out, among others, in the facilities of the Dugway Proving Ground (DPG). The device enables one to detect the concentration at the level of several hundreds to a few thousand parts per liter (ppl) from a distance of about 700 m, incorporating as small an averaging time as 1 s. Considering longer averaging times, one can arrive at the configuration capable of detecting concentration levels below 100 ppl from the distance of 1 km.

A compact ultraviolet biological trigger lidar (UBTL) instrument for the detection and discrimination of biological warfare agent (BWA) simulant aerosol clouds was developed by us [141] using a 5 mW, 375 nm semiconductor UV optical source (SUVOS) laser diode.

The system successfully passed field tests at the Dugway Proving Ground. The measuring ranges for elastic scattering were over 300 m, and for fluorescence, over 100 m. Several modifications have been made to increase the sensitivity, detection, and differentiation of particles including increasing the telescope’s collection apertures to a diameter of 200 mm, adding 266 nm and 977 nm laser emitters, and three detection channels for 266 nm and 977 nm flexible backscatter and fluorescence at 330 nm, which provided an increased operating range and sensitivity.

## 8. Summary

Fluorescence methods are the most sensitive of all the spectroscopic methods and have found wide application for the detection of bioaerosols in both civil and military applications. The instruments either use fluorescence excited by one or two wavelengths, or the entire spectrum is measured. Both steady-state and time-resolved fluorescence measurements are used. In addition to analyzers for in situ tests, lidars are also used for stand-off detection. In real-time fluorescence methods, single particles are combined in one instrument with the measurements of the size and particle shape, which reduces the number of false alarms, Measurements of air bioaerosols using UV-APS and WIBS instruments have been carried out inside various rooms (hospitals, schools) and in the natural environment of all regions worldwide, as far away as Antarctica and the Amazon forest. Several systems have also been implemented that are used by the military. These instruments are mainly used for warning, and in the case of alarms, the biological material is collected and analyzed by biochemical methods in biological field laboratories. However, they are rarely used in everyday practice. A major limitation of BW detection with the LIF method is the ability to detect only bacteria and viruses, and toxins are beyond its reach. UV LIF lidars, despite their many advantages, have a main limitation, which is the strong background signal of sunlight during the day, although the measurements are practically possible at night. In situ analyzers do not have such limitations. Advances in the construction of new light sources or sensitive matrix detectors create new possibilities for the construction of analyzers based on the excitation of fluorescence with two wavelengths and measurements of the entire spectrum, and not only the average intensity in selected spectral ranges.

In all systems using the LIF method, the most important problem is the selection of the appropriate light source. Biological materials are characterized by low fluorescence quantum yields, and therefore lasers are preferred for its excitation. Their power at a selected wavelength is orders of magnitude greater than for typical lamps, even impulse lamps (e.g., xenon). In remote detection systems, these are the only possible sources. Pulsed lasers also enable the detection of individual particles of bioaerosols. For bacteria, fungi, or plant pollen, the ideal wavelengths for fluorescence excitation are lines at 280 and 340 nm. The only lasers emitting these wavelengths are only tunable, very complicated, complex, difficult to use, and very expensive OPO and PGN. In practice, therefore, the 4th and 3rd harmonics of the Nd:YAG laser (355 and 266 nm, respectively) have been used thus far. The future is semiconductor lasers, but currently, the lowest achievable wavelength is 375 nm. The most popular WIBS system uses xenon lamps as replacements for lasers. When using them, the entire fluorescence spectrum is not measured, but only wide, selected bands. It is surprising why newer generations using LED sources have not been developed. Currently, such commercially strong, pulsed sources at 280 and 340 nm are already available, especially since the American SUVOS program is just about to build such sources for biodetection purposes.

The article presented many biodetection devices. In the vast majority, however, these are still only prototypes, which were produced in small numbers. The most common and commonly used analyzers were UV-APS and subsequent modifications of WIBS. These have played an important role in the study of aerosols around the world. Currently, how-ever, UV-APS has been withdrawn from production.

An important parameter in the detection of bioaerosols is the size of the detected particles. It is estimated that about 25% of the particles formed in the atmosphere are mi-croorganisms in sizes ranging from 0.2 μm to 50 μm. Particles with larger dimensions are practically not a threat to human health. The measurements of the sizes by scattering methods accompanying the LIF allow for the preliminary recognition of dangerous biological aerosols. Particle shape studies are of lesser importance as BW materials are a mixture of many substances. Moreover, the particles are generally stuck together and form clusters of spherical shapes. Thus, more than 90% of aerosol particles in the atmosphere are spherical in shape. Shape studies make more sense for single particles of pure bacteria or viruses.

## Figures and Tables

**Table 1:** Examples of the disease and its pathogens.

Infection	Pathogen	Diameter [µm]
Anthrax	Bacillus anthracis	1–4
Botulism	Clostridium botulinum	1–5
Plague	Vibrio cholerae	0.5–0.8
Tularemia	Francisella Tularemia	0.2–0.7
Smallpox	Mariola Major	0.15–0.3

**Table 2:** Biological sample list and their abbreviations.

No.	Name	Source	Abbreviation
Vegetative bacteria			
1	*Bacillus atrophaeus var. globigii*	ATCC 9372	BG
2	*Bacillus cereus*	ATCC 14579	BC
3	*Bacillus megaterium*	PCM 2006	BM
4	*Bacillus subtilis*	ATCC 6333	BS
5	*Bacillus thuringiensis*	ATCC 10792	BT
5	*Micrococus luteus*	ATCC 4698	ML
6	*Escherichia Coli*	ATCC 25922	EC
Endospores			
7	*Bacillus atrophaeus endospores*	ATCC 9372	BGs
8	*Bacillus cereus endospores*	ATCC 14579	BCs
9	*Bacillus megaterium endospores*	PCM 2006	BMs
10	*Bacillus subtilis endospores*	ATCC 6333	BSs
11	*Bacillus thuringiensis endospores*	ATCC 10792	BTs
Technical spores			
12	*Bacillus atrophaeus spores*	MIHE	BGst
13	*Bacillus anthracis spores*	MIHE	BAst
14	*Bacillus thuringiensis spores (TUREX)*	PVTT (Finland)	BTst
	Fungi		
15	*Alternaria alternata*	ATCC 6663	AA
16	*Candida albicans*	ATCC 18804	CA
17	*Cladosporum herbarum*	ATCC 28987	CLH
18	*Penicillium chrysogenum*	ATCC 9197	PCH
19	*Penicillium brevicompactum*	ATCC 9056	PBC
	Pollens		
20	*Bermuda grass pollen*	Duke Scientific Corp.	BER
21	*Corn pollen*	Duke Scientific Corp.	COR
22	*Paper mulberry pollen*	Duke Scientific Corp.	PAP
23	*Ragweed pollen*	Duke Scientific Corp.	RAG
24	*Johnsons Grass Smut spores*	Duke Scientific Corp.	JONs
25	*Secale cereale pollen*	Sigma-Aldrich	SEC
	Other		
26	*Ovalbumin*	Sigma-Aldrich	OVA
26	*Bacteriophage MS2*	MIHE	MS2

**Table 3:** Overview of the instruments for detecting bioaerosols.

Instrument/Method	Firm	EX [nm]	References
Laboratory system	Army Research Laboratory (ARL)	488	[34,35,80,81,82,83,84]
Fluorescence aerodynamic particle sizer (UVAPS) and military version (FLAPS)	Canadian Defense Research Establishment TSI	355	[85,86,87,88,89,90]
Biological aerosol warning system (BAWS)	MIT, Lincoln Laboratory (LL, Lexington, MA, USA)	266	[91,92,93]
Elastic scattering-cued fluorescence sensor	Naval Research Laboratory (NRL)	266	[25,94]
Rapid agent aerosol detector (RAAD)	LL NRL Edgewood Chemical and Biological Center	266 355	[15,95,96,97,98]
WIBS	BIRAL, University of Hertfordshire and Defense Science Technology Laboratories (Dstl, UK)	280	[33,78,98,99]
Verotect^TM^	BIRAL, Dstl	280	[100]
Multiparameter bioaerosol sensor (MBS).		280	[101,102]
SBS (spectral intensity bioaerosol spectrometer)		285 370	[103]
Rapid-E, (PA-300)	Plair SA, Geneva, Switzerland	337	[104,105,106,107]
BARDet (Bioaerosol detector).	Intite Optoelectronics of MUT	375	[32]

## Data Availability

Not applicable.
